# Current and future perspectives for enhancing our understanding of the evolution of plant metabolism

**DOI:** 10.1098/rstb.2024.0253

**Published:** 2024-11-18

**Authors:** Jan de Vries, Sophie de Vries, Alisdair R. Fernie

**Affiliations:** ^1^ Department of Applied Bioinformatics, University of Goettingen, Institute of Microbiology and Genetics, Goldschmidtstr. 1, Goettingen 37077, Germany; ^2^ University of Goettingen, Campus Institute Data Science (CIDAS), Goldschmidstr.1, Goettingen 37077, Germany; ^3^ Department of Applied Bioinformatics, University of Goettingen, Goettingen Center for Molecular Biosciences (GZMB), Goldschmidtstr. 1, Goettingen 37077, Germany; ^4^ Max-Planck-Institute of Molecular Plant Physiology, Am Mühlenberg 1, Potsdam-Golm 14476, Germany

**Keywords:** evolution, metabolism, specialization

## Abstract

The special issue ‘The evolution of plant metabolism’ has brought together original research, reviews and opinions that cover various aspects from the full breath of plant metabolism including its interaction with the environment including other species. Here, we briefly summarize these efforts and attempts to extract a consensus opinion of the best manner in which to tackle this subject both now and in the future.

This article is part of the theme issue ‘The evolution of plant metabolism’.

## 1. Introduction

The primary metabolism of plants is a constantly running operation that links the two major endosymbiotically gained organelles with a myriad of enzymes that, in their entirety, keep the plant cell system functioning. Here, the starkest differences are between phototrophic tissues—and its operation in the sunlight—and heterotrophic tissues (such as roots) [1–3]. The specialized metabolism of plants on the other hand is an ebb and flow that is foremost determined by environmental factors [4–6]. Combined with enzyme promiscuity and the direct action of abiotic factors (e.g. inducing oxidative cleavage), this creates a hotbed of the evolutionary emergence of chemodiversity—and eventual evolutionary fixation of genes coding for enzymes that catalyse the formation of potent bioactive compounds [7–11]. In this special issue, diverse aspects behind the evolutionary emergence of chemodiversity have been investigated, synthesized and discussed.

## 2. Advancements in studying the evolution of plant metabolism

Ancestral sequence reconstruction (ASR), also known as ancestral gene construction, is an approach that provides statistical estimates of ancient sequences allowing them to be resurrected and in the case of enzyme-encoding genes kinetically characterized in the laboratory. In his visionary article, Barkman describes the utility of this technique in plants, where much of the seminal work was performed by his own lab [12]. He details how studies using this approach revealed that exaptation is commonplace such that ancestral enzymes often cannot catalyse the reaction that becomes the prime activity of its descendent enzymes. He also argues that intramolecular epistasis may or may not limit evolutionary paths towards switches in catalytic or substrate preferences, and finally postulates that ancient pathway flux is likely highly divergent from that of modern-day metabolic networks. In a second technically oriented article, Naake *et al*. presented a combined phylogenetic and phylogenomic syntenic analysis based on whole-genome sequences of 126 species spanning Stramenopiles and Archaeplastida including *Arabidopsis thaliana*, *Solanum lycopersicum* and *Zea mays*, thereby combining the study of genomic location with changes in gene expression [13]. Their study revealed that serine carboxypeptidase (SCP)/SCP-like (SCPL) family genes have a deeper evolutionary origin than BAHD family genes as well as differences in their evolutionary history. However, they also demonstrate the current limitations of this approach being unable to separate authentic SCPL acyltransferases from the more standard SCP peptidyl hydrolases. The final technically oriented article in this collection, that by Lee *et al*., focuses on transcription factor analyses. In their article, they provide compelling arguments for exploring evolutionary relationships between transcription factors and their targets as a powerful tool for selecting the best transcription factor for a given metabolic engineering strategy [14]. Lee *et al*. argue that, in this respect, learning from the past is a better indicator than protein sequence homology alone.

Following these three methods-oriented articles, we clustered together a set of papers that broadly focus either on general aspects of the evolution of plant metabolism (or of physiology) and how it is interwoven with general aspects of plant biology.

## 3. Plant physiology interplaying with a chemodiverse specialized metabolism

Plant microbe interaction builds on an ancient molecular chassis [15–18]. The article by de Vries & Feussner [19] discusses how biotic interactions are major drivers of lineage-specific metabolic diversification. Their aim is to solicit evolutionary forces with both the micro- and macro-evolutionary diversifications in mind. De Vries & Feussner [19] argue that the core of many ‘land plant-specific’ pathways for biotic interactions are present in algae, for which several examples are a case in point [20]. The specific evolutionary ramifications are, however, so they argue, likely complex because of a multitude of input and output from different species. Here, possible environment-dependent metabolic plasticity could be an opportunity for rapid adaptation and, from a macro-evolutionary point of view, together with noise and enzyme promiscuity lead to diversification of metabolism. Indeed, de Vries & Feussner [19] make the point that specialized metabolism must be plastic—it cannot be selected for a limited function, as it pertains to (i) the ability to quickly respond to changing environments, (ii) being implemented in multiple processes, and (iii) being robust upon modulation by pathogens or side products of degradation processes in microbial interactions. Hence, selection towards diversity can be key for the evolutionary history of specialized metabolism. This may be mirrored in a macro-evolutionary outcome, possibly explaining the recently identified metabolic higher diversity of previously announced conserved metabolic processes [19], as exemplified by the realization of diverse phytohormone roles and bioactive ligands [21–27].

The article by Abel and Nauman describes how access to vital mineral nutrients profoundly affects growth development and vigour thereby setting limits on primary productivity in natural ecosytems and crop production in modern agriculture, respectively [28]. They use genomic evidence to trace the chemical evolution of phosphate, the geochemical phosphorus cycle and its acceleration during the Anthropocene as well as during the evolution and rise of terrestrial plants focusing on phosphate mobilization and acquisition. Beyond this, they discuss how the activities of mankind have impacted on the global phosphorus cycle and profiling. Key to the phosphate acquisition of land plants are arbuscular mycorrhizal symbioses. In their article, Delaux and Gutjahr examine the arbuscular mycorrhizal symbioses formed by most extant land plants with fungi and which evolved 450 million years ago [29]. They highlight how, although several plant genes leading to an efficient symbiosis have been identified, our understanding of the metabolic processes involved remains limited. That said, the exchange of chemical signals, a number of dedicated biosynthetic pathways and specialized metabolites involved in late stages of the symbiosis are starting to be uncovered. Finally, Delaux and Gutjahr propose routes by which phylogenetic tools can be used to assess the roles played by small molecules in ancient plant symbiosis.

Carotenoids are pivotal pigments of land plants and algae owing to their accessory functions in the photosystem complexes. They are key for environmental adaptation and acclimation. And indeed, photosynthesis-associated responses upon environmental perturbance are among the clearest conserved patterns gleaned from investigations even on the close relatives of land plants [30,31]. Yet, this role alone does not do their diverse functions justice, as they serve as starting points for further catabolism and modification [32]. This yields diverse specialized metabolites that include apocarotenoids that emerge through oxidative cleavage [32,33]. The type and diversity of compounds that can emerge from carotenoids depends on modification of the primary carotenoid as well as modification of its catabolite, cleavage product and so on. Rieseberg *et al.* [34] have investigated a peculiar case of red, carotenoid-based, pigmentation in a species of Charophyceae, *Chara tomentosa. C. tomentosa* is a macrophytic alga of several dozen centimetres height that accumulates a vibrant red pigmentation not only in its antheridia but throughout its body [35]. Now, Rieseberg *et al*. [34] found that these plants not only hyperaccumulate the monocyclized carotenoids δ-carotene and γ-carotene but also exude a rich bouquet of volatile apocarotenoids. This warrants attention since both in previously published data [36,37] and in their new transcriptome of *C. tomentosa* [34], no homologs of *carotenoid cleavage dioxygenase* (*CCD*) were found. CCDs catalyse the enzymatic formation of apocarotenoids [38]. Thus, the presence of apocarotenoids in *C. tomentosa* suggests massive non-enzymatic oxidative cleavage or alternative enzymatic routes [34]. Regarding the latter, Rieseberg *et al*. [34] found high expression of a cytochrome P450 enzymes homologous to MORE AXILLARY BRANCHES 1 (MAX1) coinciding with high apocarotenoid levels. MAX1 is a key for the biosynthesis of some of the most famous apocarotenoids, the strigolactones [39]. This raises the interesting possibility that a diversified apocarotenoid metabolism occurs in *Chara*, building on yet-to-be-discovered biosynthetic routes with, as yet, unknown biological functions.

Along these lines, Alvarez *et al*. [40] have characterized a β-carotene isomerase of a cyanobacterium that is homologous to the DWARF27 (D27) enzyme. The enzyme D27 is known from land plants as the first committed step in strigolactone biosynthesis [41]. Alvarez *et al*. [40] found that the homolog they characterized from *Cyanobacteria aponinum* catalyses divergent reactions (e.g. signified by different isomeric preferences). The enzyme, however, accepted different carotenes as substrate, and the authors concluded that it likely functions in the maintenance of the *cis*-β-carotene pool. Overall, this cyanobacterial D27 is a prime example of how much chemodiversity, building on the same homologs (albeit very divergent), can be found across various branches on the tree of life. Simply put, more phylodiverse sampling to discover the possible enzymatic activities (enzyme space) is needed.

Autophagy is a eukaryote-wide mechanism [42] that acts in selective digestion of cellular components to accrue resources and remove dysfunctional components. The article by Laude *et al*. [43] discusses that while autophagy is a key mechanism in the response of diverse photosynthetic eukaryotes to fluctuating conditions (which particularly impact them), we know very little about the molecular mechanisms outside of a few model systems. By tracing the evolutionary history of key genes and outlining the physiological relevance as well as regulation of autophagy, Laude *et al*. [43] provide a guideline for future research into the diversity of autophagy.

As the reader will have noted, this special issue is largely devoted to specialized metabolism given that it is the more phylogenetically divergent. That said, important aspects of papers covering areas of the evolution of primary metabolism which we felt were underrepresented in prior studies, namely fatty acid biosynthesis, vitamin B12, glutathione conjugates and aromatic amino acid metabolism, are also representative of the massive advances in our understanding that have been brought about by the torrent of genomic data produced over the last decade or so. In the first of these articles, Conrado *et al*. provided an overview of the evolutionary importance of the committed step for de novo fatty acid biosynthesis—that catalysed by acetyl CoA carboxylase (ACCase) [44]. They analysed protein–protein and co-expression networks in *A. thaliana* and *Chlamydomonas reinhardtii* suggesting intricate associations with ACCase. Conrado *et al*. further investigated the extent to which genes involved in the ACCase regulatory network arose in autotrophic eukaryotes providing first insights into their evolutionary trajectory. The second type of primary metabolites covered are the vitamins with Dorrell *et al*. providing a comprehensive review of vitamin B-cobalamin [45]. This review surveys the alternative routes for the synthesis and consequences of its availability for enzyme performance. Intriguingly, considering the collection sites of algae that have lost B12 metabolism, they propose that freshwater-to-land transitions (also covered in [16]) and symbiotic associations (also covered in [29]) have constrained B12 availability in early plant evolution. In the third article of this set, Nikola *et al*. focus on glucosyl transferases [46]. While it is clearly at the crossroads of primary and specialized metabolism, glutathione, which is the hub metabolite, has its roots as a primary metabolite. In their article, Nikola *et al*. clearly state that a problem in our current understanding is that the endogenous glutathione conjugates produced by these enzymes are rarely reported. They go on to detail how high substrate promiscuity and the fact that many of the substrates are prone to spontaneous chemical reactions further complicate their study. Despite these problems, however, Nikola *et al*. summarize the available knowledge concerning these important enzymes attempting to define how and why evolution has resulted in such an extensive expansion of this gene family. In the fourth primary metabolism related article, Yokoyama describes the evolution of aromatic amino acid biosynthesis and usage as precursors for plant natural product biosynthesis [47]. He showcases the intra- and inter-kingdom diversity and origin, or the committed enzymes involved in plant aromatic amino acid biosynthesis and catabolism, providing a perfect bridge to the articles focused on phenylpropanoids that follow.

## 4. Phenolics

Land plants have interwoven biochemical pathways that are best described as networks [8,48]. Their chemodiverse phenolics are a point in case. Kunz *et al*. [49] explored the question of how these came about, with a focus on the roles that shikimate-pathway-derived phenolics—which include those that funnel into and from the phenylpropanoid pathway—have in environmental response. Here, the deep evolutionary origin of streptophytic stress-responsive phenolics that likely emerged in a last common ancestor (see [20] and [50]), which lived at a time long before land plants came about, was highlighted [49]. Furthermore, Kunz *et al*. [49] conceptualized how convergent and parallel evolution can shape an effective cocktail of phenolics that has stress-adaptative properties; this treatise helps to explain the recurrent evolution of complex phenolic profiles. A prime case of this complexity are the flavonoids. Davies and colleagues [51] explore the evolutionary origin of flavonoids—the most chemodiverse group of specialized phenolic compounds. Also here, the possible deep evolutionary origin in streptophyte algae is highlighted (and detection of flavonoids has been reported for algae, e.g. refer to [52] and [53]), with the role of environmental responses being further highlighted. The authors make a strong argument for pushing functional work outside of the few land plant systems and consolidation of efforts for understanding the physiology and regulation of flavonoids across streptophyte diversity.

Streptophyte algae have become an important reference point for many comparative studies that focus on the evolution of the unique biochemistry of land plants. Yet, they have exiting and unique biology in their own right. The closest algal relatives of land plants are the extremely diverse and species-rich Zygnematophyceae that fall into five deeply divergent orders [54–57]. Zygnematophyceae are united by a unique mode of reproduction, conjugation that results in intricately structured zygospores [58,59]. Permann & Holzinger [60] have provided a detailed account on the complex amalgamate of lipid droplets (likely a key synapomorphy of zygnematophyceaen algae and embryophytes [61]), phenolics and layered cell walls. Shedding light on how these structures are made and function is key for our understanding of the complex trait of resilient and dormant structures that unite endurance and propagation.

Agorio *et al*. [62] examine the current state of knowledge on specialized metabolism during plant–microbe interactions across a wide range of phylogenetic lineages. In their article, they consolidate not only information from the literature but also wade through genomic, metabolomic and transcriptomic data to pinpoint specific responses. For this, they focus on the deep evolutionary roots of phenylpropanoid biosynthesis, which is likely an ancient traits of (at least) phragmoplastophytes and Klebsormidiophyceae [20] and involved in the response to pathogen attack also in non-vascular plants [63]. The authors highlight the dynamic history of secondary metabolism, and point out that the gains, expansions and also losses of certain routes and final products must have shaped the interactions of host plants with their pathogens. Overall, this begs the question of how deep the role of phenylpropanoid-derived metabolites in biotic responses traces in streptophyte evolution and highlights the need for a closer look into alternative and possible lineage-specific pathways to fully understand the evolution of the metabolic immune system of land plants.

While arguably not as well studied as the phenylpropanoids, the other articles in this collection focus on other areas of specialized metabolism.

## 5. Evolution of chemodiversity

The chemodiversity of land plants’ specialized metabolism impresses and puzzles in equal measure. How this generally came about is at the heart of the paper by Ji *et al*. [64]. First of all, they highlight gene duplications—which is a powerful source of novelty, particularly in plants [65], allowing for re-contextualization of existing functional domains [66] evolution of the resulting sequences under relaxed constraints and thus allowing for neofunctionalization. Second, Ji *et al*. [64] discuss promiscuity, which provides a background level of metabolic noise created by enzymes on which evolution can act [8,67]. Finally, the relevance and increasing recognition of the evolution of plant biosynthetic clusters are highlighted [64,68]—which are a great means of finding conserved biosynthetic modules across macroevolutionary scales [69].

One of the most diversified group of enzymes involved in specialized metabolism are the cytochrome p450 (CYP) proteins [70–74]. Werck-Reichhart *et al*. [75] highlight the deep conservation of CYP proteins in diverse Archaeplastida. They, however, further stress that in streptophytes and especially land plants, CYPs diversified and thus gave rise to the CYP families that underpin important biopolymer and signalling functions. They stress that these contribute to various traits that are adaptive in a terrestrial context. These include polymers [58,76–78] that increase the resilience of plant (and algal) structures by enhancing water retention, UV protection and more [75]. Finally, Werck-Reichhart *et al*. [75] stress that CYPs are involved in the production of diverse signalling molecules, including the aforementioned CYP enzyme MAX1 [39], for strigolactones but also the gibberellins. The deep evolution of gibberellins as signalling molecules is currently enigmatic and rather hinges on related molecules [79–83], but it clearly builds on the action of CYPs.

The article of Jia *et al*. focuses on purine biosynthesis in plants [84]—interestingly one of the key pathways investigated by the ancestral sequence restriction approach described by Barkman [12]. Purine alkaloids have arisen via convergent evolution in a wide range of species. In their review, Jia *et al*. detail the metabolic processes and their regulation alongside evolutionary insights that have arisen from genome and transcriptome sequencing. They highlight how these studies have brought great insight into elucidating inter-species interactions and adaptive evolution as well as discussing the ecological roles of purines in various plants. Similarly, the article of Olivia *et al*. focuses on sesquiterpene lactones, a group of metabolites found predominantly in the Asteraceae family with multiple ecological roles and medicinal applications [85]. It describes the known links between the chemistry of intermediates of this pathway and the modifications which contribute to their massive chemical diversity. Finally, they also describe methods for identifying and predicting the pathways underlying sesquiterpene lactone biosynthesis thereby illustrating the enormous, but as yet not fully tapped potential, of genome- and transcriptome-based computational analysis of metabolism.

The articles collected within this special issue not only provide a representative update of the currently ongoing research concerning the evolution of plant metabolism (and physiology) but also aid in drawing a roadmap for the future study of the evolution of metabolism which will greatly expand upon the state-of-the-art as presented in the opening article [86]. They have shed light on the mechanisms that have driven metabolic innovations, a combination of the genetic and environmental factors that have shaped the metabolic networks of diverse plant species. Moreover, they offer a comprehensive examination of how these metabolic pathways have adapted to various ecological niches, thereby enhancing our understanding of plant resilience and adaptability. Here the advancements made in establishing a robust phylogenetic framework [54,87–96] and genomic sampling covering now at least one representative from almost all major lineages of streptophytes [37,53,97–105] as well as phylodiversity outside of streptophytes [106–115] are critical for our ability to sample the capabilities of streptophyte enzymes and the biosynthesis routes they shape. For example, lycophytes have evolved a unique solution for lignin biosynthesis [101,116–120], and the routes for lignin biosynthesis in ferns appear again different from both angiosperms and lycophytes [20,120]. Such cases warrant attention, as they pave the way for novel biotechnological applications aimed at optimizing plant metabolism for improved agricultural productivity and sustainability.

One of the postulates raised by Barkman [12]—that ‘ancient pathway flux is likely highly divergent from that of modern-day metabolic networks’—would be intriguing to test. Simple comparisons between extant species that emerged at different timescales would represent a first step in this direction. It is worth noting that since the conception of this special issue ASR has been expanded from single genes to the genome level [121,122], hence, it is conceivable that a more exact comparison may be possible in the future. The observation that combining phylogenetic and syntenic analyses does not allow the discrimination between authentic SCPL acyltransferases from SCP peptidyl hydrolases suggests that selective functional analyses will likely greatly improve the power of this approach. The idea of Sun Yun *et al*. that understanding the evolutionary trajectory of transcription factors can enhance metabolic engineering prospects is a powerful one and it will be interesting to see if this proves to be true at scale.

Also, several current articles, such as Davies *et al*. [51] and Werck-Reichhart *et al*. [75], and past ones [8] have highlighted the role of the specialized metabolism as a massive carbon sink. This is particularly interesting in the context of the biogeochemical changes in carbon availability throughout evolution. Experimental evolution that combines engineered pathway flux applying different CO_2_ conditions would be intriguing.

The collection of articles presented here highlights that genomic and transcriptomic sequencing, and to a lesser extent metabolomics, have played massive roles in enhancing our understanding of the evolution of plant metabolism. While these techniques have addressed a wide range of evolutionary questions their scope is as yet relatively untapped and expanding their use for other pathways and/or gene families will likely continue to elevate our understanding in the next few decades. This greater use of computational tools alongside more frequent characterization of enzyme space represent important tools, which will undoubtably be essential in complementing such studies. Among these, the use of machine learning in protein structure/function studies will almost certainly be highly important. In this vein, the co-evolution of proteins and their small molecule ligands will be highly informative in both metabolic and signalling functions alike [123].

## Data Availability

This article has no additional data.
